# Flavonifractor plautii: A Rare Cause of Anaerobic Bacteremia

**DOI:** 10.7759/cureus.86478

**Published:** 2025-06-21

**Authors:** David Zamora Diaz, Sergio Llanes Somellera

**Affiliations:** 1 Internal Medicine, Loyola University Medical Center, Berwyn, USA

**Keywords:** anaerobic infection, bacteremia, diarrhea, flavonifractor plautii, immunocompromised host

## Abstract

*Flavonifractor plautii* (formerly *Eubacterium plautii*) is a Gram-variable, strict anaerobic bacillus that forms part of the human gut microbiome, although its role in human health remains incompletely understood. *F. plautii* has rarely been linked to clinical infections. We describe a case of bacteremia in a patient with severe intrahepatic biliary ductal dilatation and septic shock and *F. plautii* bacteremia, which cleared following treatment with ceftriaxone and metronidazole. Diarrhea with disruption of the gut barrier and subsequent bacterial translocation appear to be the mechanism for bacteremia due to this organism. Based on this case and previous reports, *F. plautii* infections appear susceptible to beta-lactam antibiotics (e.g., ampicillin, ceftriaxone) and anaerobic agents (e.g., metronidazole, clindamycin), supporting their use as preferred treatment options over glycopeptides or oxazolidinones. This case underscores the importance of recognizing anaerobic gut commensals as potential bloodstream pathogens in critically ill patients.

## Introduction

*Flavonifractor plautii* is a Gram-variable, strict anaerobic bacillus. This species is part of the human gut microbiota, belonging to the order Eubacteriales, family Oscillospiraceae, and is the type species of the genus Flavonifractor. It demonstrates reduced susceptibility to glycopeptides and oxazolidinones, such as vancomycin and linezolid [1]. While it is a commensal member of the human gut microbiome, its clinical significance is not fully understood. Emerging research suggests both potential health benefits, such as protecting against arterial stiffness [2] and attenuating inflammation [3], whereas others have linked its presence to an increased risk of young-onset colorectal cancer [4]. Reports of *F. plautii* as a cause of bacteremia and other infections remain exceedingly rare, with only 10 cases described worldwide to date, to our knowledge [5-14].

## Case presentation

We report a case of a 75-year-old woman with a past medical history of atrial fibrillation on apixaban, hypertension, chronic obstructive pulmonary disease with emphysema, heart failure with reduced ejection fraction, and chronic alcohol use, who presented to the emergency department with hypothermia with a core temperature of 32.6 °C, hypotensive with a blood pressure of 82/45 mmHg, and tachycardic at 118 beats per minute. She was tachypneic with a respiratory rate of 26 breaths per minute and had an oxygen saturation of 91% on room air. Her mental status was significantly altered, with a Glasgow Coma Scale (GCS) score of 10, prompting endotracheal intubation for airway protection.

Initial laboratory studies revealed a white blood cell count of 16,500/µL (reference range: 4,000-10,000/µL). Hemoglobin was 7.8 g/dL (reference range: 12-16 g/dL). Platelets were decreased at 75,000/µL (reference range: 150,000-400,000/µL). Serum lactate was elevated at 5.2 mmol/L (reference range: 0.5-2.0 mmol/L). Renal function was impaired, with a creatinine of 2.8 mg/dL (baseline 0.9 mg/dL; reference range: 0.6-1.3 mg/dL), and blood urea nitrogen (BUN) was elevated at 58 mg/dL (reference range: 7-20 mg/dL). Liver-associated enzymes showed mild transaminitis (AST 98 U/L, ALT 74 U/L; reference range: 10-40 U/L), an elevated total bilirubin of 3.6 mg/dL (reference range: 0.1-1.2 mg/dL), and an alkaline phosphatase level of 312 U/L (reference range: 44-147 U/L). Arterial blood gas revealed a pH of 7.21 and bicarbonate of 16 mmol/L (reference range: 22-28 mmol/L), indicating a primary metabolic acidosis. Electrolytes showed mild hyponatremia (Na 133 mmol/L; reference range: 135-145 mmol/L), normal potassium (K 4.2 mmol/L; reference range: 3.5-5.0 mmol/L), chloride of 100 mmol/L (reference range: 98-106 mmol/L), and low serum bicarbonate of 15 mmol/L (reference range: 22-28 mmol/L).

**Figure 1:**
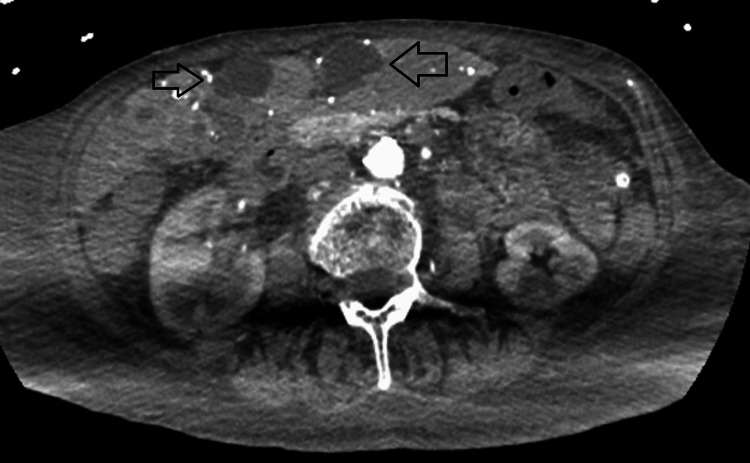
Computed tomography of the abdomen showing hepatic hypodense lesions (black arrows)

**Figure 2:**
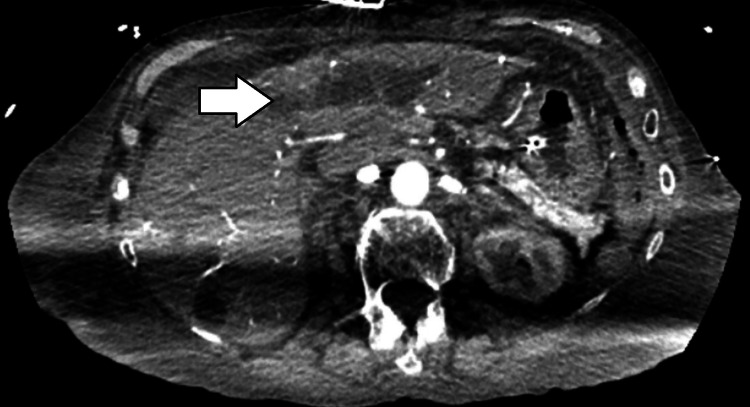
Computed tomography of the abdomen showing left biliary ductal dilation (white arrow)

On initial evaluation, a computed tomography (CT) scan of the abdomen revealed numerous hypodense hepatic lesions (Figure 1), severe biliary ductal dilatation within the left hepatic lobe (Figure 2), extensive third-spacing, signs of colitis with bowel edema, and a non-visualized gallbladder, raising concern for biliary pathology. 

Blood cultures obtained on admission grew *F. plautii* (1 of 2 bottles), reported on hospital day 5. Initial Gram staining revealed Gram-negative rods; however, subsequent review demonstrated variable staining with Gram-positive features, confirmed by Bruker MALDI-TOF MS. Antimicrobial susceptibility testing was not performed as *F. plautii* isolate was detected on hospital day 6, while repeat blood cultures obtained on day 3 remained negative. Given the clinical improvement with empirical treatment and the negative repeat cultures, susceptibility testing was not standard practice for rare or atypical isolates unless explicitly requested, and the decision was made to prioritize clinical management. The clinical course, including the resolution of bacteremia with empirical therapy, supported this decision. 

The patient had reported diarrhea prior to admission, but investigations for its causative agent, including *Clostridium difficile*, were negative. The patient was initially treated with ceftriaxone and metronidazole from hospital days 1 to 3. Vancomycin was added for MRSA pneumonia coverage (hospital days 1 to 7). Given clinical deterioration, antibiotic therapy was broadened to piperacillin-tazobactam on hospital day 3 and continued through hospital day 10. Subsequent blood cultures remained with no growth; however, the patient's overall clinical course was complicated by multiorgan failure, and she expired on hospital day 14. 

## Discussion

Although *F. plautii* is a strict anaerobe, bacteremia can occur, particularly in patients who are critically ill or immunosuppressed. One of the key mechanisms underlying bacteremia with *F. plautii* and other anaerobes is bacterial translocation, which can occur when gastrointestinal integrity is compromised. Acute illness induces a systemic inflammatory response that can lower tissue oxygen levels through microvascular dysfunction, impaired perfusion, and increased oxygen consumption at sites of inflammation. Evidence shows that many anaerobic bacteria possess substantial oxidative stress defenses and can transiently withstand low levels of oxygen exposure [15].

Literature search methodology

A structured literature search was conducted using PubMed to identify reported cases of *Flavonifractor plautii *or *Eubacterium plautii *infections. The search was limited to English-language articles only, with no restrictions on publication date.

Two primary search strategies were used: ("Flavonifractor plautii"[Supplementary Concept] OR "Flavonifractor plautii"[All Fields] OR "Eubacterium plautii"[All Fields]) AND ("infect"[All Fields] OR "infection"[All Fields] OR "infections"[MeSH Terms] OR "pathogenicity"[MeSH Subheading] OR related infection terms). This search yielded 16 results. "Flavonifractor plautii"[All Fields] AND "infection"[All Fields]. This yielded 14 results.

All articles were screened manually. Inclusion criteria were reports describing human infection attributed to F. plautii or E. plautii. Cases where the organism was isolated from sterile sites, such as blood, cerebrospinal fluid, pleural fluid, or joint fluid. Exclusion criteria included experimental or animal studies, reports without clear attribution of infection to F. plautii, and abstracts without full text available in English. Ultimately, 10 relevant case reports of bloodstream infections met inclusion criteria and were summarized in the manuscript.

Table 1 summarizes our findings and compares the various presentations of bacteremia due to *F. plautii. *Reported cases commonly associate bacterial translocation with gastrointestinal disturbances, such as infectious colitis, diarrhea, and conditions like ileal perforation requiring laparotomy, which allow the migration of anaerobic organisms from the gastrointestinal tract to the bloodstream.

**Table 1:** Summary of reported cases of Flavonifractor plautii bacteremia, associated clinical features and therapeutic approaches

Case	Age/Sex	Primary disease and Risk factors	Diarrhea present	Gram Stain	Therapy Used	Outcome
Garre et al. [5]	35/Male	Dog bite. History of splenectomy	Yes	Negative rods	Benzylpenicillin	Recovered from bacteremia
Berger et al. [6]	69/Male	Acute cholecystitis	Yes	Positive rods	Ceftriaxone and metronidazole	Recovered from bacteremia
Karpat et al. [7]	24/Female	Infectious colitis. History of splenectomy	Yes	Not available information	Meropenem and metronidazole	Recovered from bacteremia
Costescu et al. [8]	45/Male	Diarrhea	Yes	Culture plate: negative rods	Amoxicillin- clavulanate	Recovered from bacteremia
Wilton et al. [9]	62/Male	Diarrhea	Yes	Variable rods	Ceftriaxone	Recovered from bacteremia
Saito et al. [10]	83/Male	Stenosis in the transverse colon and generalized peritonitis. History of gastric cancer, and hepatocellular cancer	Yes	Negative rods	Piperacillin/ tazobactam	Recovered from bacteremia
Osada et al. [11]	75/Male	Inflammatory bowel disease unclassified and Clostridioides difficile colitis	Yes	Blood culture fluid: negative rods, Culture plate: positive rods	Levofloxacin and metronidazole	Recovered from bacteremia
Chen et al. [12]	46/Male	Obstructive nephropathy and abdominal infection. History of cervical cancer	Not available information	Negative rods	Piperacillin/ tazobactam and moxifloxacin	Recovered from bacteremia
Orlando et al. [13]	33/Male	Febrile neutropenia transplant recipient	No	Variable rods	Not available information	Recovered from bacteremia
Kvernberg et al. [14]	48/Female	Ulcerative colitis and anal cancer	Not available information	Not available information	Meropenem and metronidazole	Recovered from bacteremia
This case	75/Female	Biliary duct dilatation, unspecified hepatic lesions	Yes	Blood culture fluid: negative rods, Culture plate; positive rods	Ceftriaxone and metronidazole	Recovered from bacteremia

At least 8 out of 11 reported cases of *F. plautii* bacteremia, including ours, involved patients who presented with diarrhea [5-11]. As observed in our case and others, diarrhea is a significant clinical feature that may facilitate the process of bacterial translocation. This mechanism was observed in other cases of bacterial translocation, such as in patients with inflammatory bowel disease, Clostridioides difficile colitis, or stenosis of the transverse colon, all of which share a disrupted gastrointestinal tract as a common risk factor for bacteremia. 

In our patient, gastrointestinal disruption with colitis and intrahepatic biliary dilatation may have played a crucial role in the translocation of *F. plautii*. The case adds to the growing body of literature that supports the role of gastrointestinal dysfunction, particularly diarrhea, as a key player in the pathogenesis of bacterial translocation, especially in immunocompromised patients, accounting for 7 out of 10 cases [5,7,10-14]. The association between gastrointestinal symptoms and bacteremia caused by anaerobic pathogens should be considered when diagnosing similar cases. 

*F. plautii *was initially isolated from only one of the two blood culture bottles, with the other culture bottle being negative, possibly due to the inherent difficulties in growing strict anaerobes. Moreover, given the lack of current evidence that *F. plautii* could be isolated in the skin, its detection in blood cultures should be considered significant rather than a contaminant, especially in the context of gastrointestinal infections, risk factors including immunosuppression, signs of systemic infection, and absence of other pathogens, supports a true bacteremia.

Moreover, some of the published reports of *F. plautii *infection also describe isolation from a single anaerobic bottle, reflecting the inherent difficulty in culturing fastidious anaerobes. This is particularly important for obligate anaerobes, as they usually reflect a true bacteremia, even if it is isolated on a single bottle [16,17].

Additionally, the Gram staining of the organism was initially Gram-negative, then later Gram-positive rod forms, which is characteristic of *F. plautii* due to potential cell wall alterations after oxygen exposure [1]. 

Regarding treatment, across published case reports, in the study by Saito et al., *F. plautii *demonstrated consistent susceptibility to all beta-lactams tested and metronidazole, while showing resistance to clindamycin, macrolides, highlighting the need for targeted anaerobic coverage guided by susceptibility testing [10]. This supports the use of beta-lactams (e.g., ceftriaxone) [6-11,13,15] and anaerobic agents (e.g., metronidazole) [6,7,11,14] as effective treatments and highlights the limited role of glycopeptides and oxazolidinones due to intrinsic resistance or reduced susceptibility in managing this infection. In our case, while blood cultures cleared and the patient improved transiently, the overall clinical outcome was poor, and thus conclusions about treatment efficacy should be interpreted with caution.

## Conclusions

In conclusion, this case highlights the potential for *F. plautii*, a gut commensal and obligate anaerobe, to cause bloodstream infection in critically ill patients, particularly those with gastrointestinal disruption, diarrhea, and possible biliary tract pathology. The growing number of reports, including ours, consistently describe patients presenting with diarrhea and gastrointestinal barrier disruption, with infection attributed to bacterial translocation from the gut. This suggests a pattern of translocation-related bacteremia in immunocompromised or acutely ill individuals. Recognizing this link is vital not only for timely diagnosis but also for guiding empiric antimicrobial therapy and informing broader clinical management strategies in complex, high-risk patients.

Our patient's bacteremia resolved with empirical treatment using beta-lactams and anaerobic agents, which aligns with prior cases and supports these as reasonable therapeutic choices. However, this case also underscores important limitations: antimicrobial susceptibility testing was not performed, and the organism was isolated from only one of two blood culture bottles. These factors limit the generalizability of the findings and warrant caution in drawing broad conclusions. Further clinical experience and microbiological data are needed to better define the pathogenic potential and optimal management of *F. plautii* infections.
